# Intracranial arterial stenosis in Caucasian versus Chinese patients with TIA and minor stroke: two contemporaneous cohorts and a systematic review

**DOI:** 10.1136/jnnp-2020-325630

**Published:** 2021-03-30

**Authors:** Xinyi Leng, Robert Hurford, Xueyan Feng, Ka Lung Chan, Frank J Wolters, Linxin Li, Yannie OY Soo, Ka Sing Lawrence Wong, Vincent CT Mok, Thomas W Leung, Peter M Rothwell

**Affiliations:** 1 Nuffield Department of Clinical Neurosciences, University of Oxford, Oxford, Oxfordshire, UK; 2 Department of Medicine & Therapeutics, The Chinese University of Hong Kong, Hong Kong, China

## Abstract

**Background:**

Intracranial arterial stenosis (ICAS) is an important cause of stroke worldwide. Separate reports in Caucasians and Asians with stroke/transient ischaemic attack (TIA) have suggested lower ICAS prevalence in Caucasians, but there has been no direct comparisons of the two ethnic groups with the same criteria to define ICAS.

**Methods:**

Acute minor stroke or TIA patients in two cohorts respectively recruiting patients in Oxford (2011–2018, predominantly Caucasians) and Hong Kong (2011–2015, predominantly Chinese) were compared. ICAS was defined as ≥50% stenosis/occlusion in any major intracranial artery in MR/CT angiography. Prevalence, distribution and risk factors of ICAS were compared between the two cohorts. We also systematically reviewed literature on ICAS prevalence in stroke/TIA patients in different populations.

**Results:**

Among 1287 patients from Oxford and 691 from Hong Kong (mean age 69 vs 66), ICAS prevalence was higher in Chinese than in Caucasians (43.0% vs 20.0%; OR 3.02; 95% CI 2.47 to 3.70; p<0.001), independent of age (age-adjusted OR 3.73; 95% CI 3.00 to 4.63; p<0.001) and vascular risk factors (multivariable-adjusted OR 3.21; 95% CI 2.56 to 4.02; p<0.001). This ethnic difference was greater (p interaction=0.005) at age <70 years (OR 5.33; 95% CI 3.79 to 7.50; p<0.001) than at ≥70 years (OR 2.81; 95% CI 2.11 to 3.74; p<0.001). ICAS prevalence increased with age and with vascular risk factors in both cohorts, with equivalent prevalence in Chinese aged <60 years and Caucasians aged ≥80, and in Chinese with no vascular risk factor and Caucasians with two vascular risk factors. ICAS locations also differed between Chinese and Caucasian patients.

**Conclusions:**

Chinese are more susceptible to ICAS than Caucasians, with an earlier onset age and a higher prevalence, independent of vascular risk factors.

## Introduction

Intracranial arterial stenosis (ICAS), predominantly atherosclerotic, is an important cause of ischaemic stroke worldwide.^1^ Patients with minor stroke or transient ischaemic attack (TIA) with ICAS face a high risk of recurrence despite timely medical treatment; for instance, minor stroke or TIA patients with ICAS had a significantly higher risk of recurrent stroke within 90 days than those without ICAS (12.5% vs 5.4%, p<0.001; HR 2.39, 95% CI 1.57 to 3.66, p<0.001), treated within 24 hours with dual or mono antiplatelet therapies in the Clopidogrel in High-Risk Patients with Acute Non-disabling Cerebrovascular Events (CHANCE) trial.^2^

Indirect comparisons of separate studies have suggested large ethnic differences between Caucasians and Asians in ICAS prevalence among both TIA/ischaemic stroke patients and asymptomatic populations, with significantly higher ICAS prevalence in Asians.^1 3^ However, these studies were heterogeneous in the recruitment period, subjects (eg, mean age, stroke severity) and in imaging modalities/methods to define ICAS. To our best knowledge, there has been no direct comparison between Caucasians and Chinese in the prevalence and risk factors of ICAS in TIA/stroke patients, based on data from contemporaneous cohorts using the same imaging modalities and the same criteria to define ICAS.

To compare the risk factors of ICAS in Caucasians and Asians, to estimate the yields of routine imaging of cerebral arteries, and to understand the impact of imaging on ischaemic stroke subtyping, it is important to reliably determine these ethnic differences and the extent to which they are independent of age and vascular risk factors. Therefore, in the current study, we compared two contemporaneous cohorts of acute TIA and minor stroke patients recruited in Oxford, UK (predominantly Caucasian) and in Hong Kong SAR, China (predominantly Chinese) using MR angiography (MRA) and/or CT angiography (CTA) for the diagnosis of ICAS. We also systematically reviewed existing literature on ICAS prevalence in TIA and ischaemic stroke patients in different populations around the world.

## Methods and materials

### Subjects

In a cross-sectional study, patients with acute TIA or minor ischaemic stroke with brain MRA/CTA at baseline from two prospective, contemporaneous cohorts were investigated: all eligible patients enrolled in Oxford Vascular Study (OXVASC) from January 2011 to December 2018, and all eligible patients admitted within 24 hours of symptom onset enrolled in the Chinese University of Hong Kong Stroke Registry (CUHK-SR) from January 2011 to December 2015. TIA was defined as a transient episode of neurological dysfunction caused by focal brain or retinal ischaemia that completely resolved within 24 hours. Minor ischaemic stroke was defined as sudden onset of neurological deficits caused by brain ischaemia lasting longer than 24 hours, with a National Institutes of Health (NIH) Stroke Scale (NIHSS) ≤3.

OXVASC is an ongoing population-based study recruiting all patients with acute vascular events (including TIA and ischaemic stroke; predominantly Caucasian patients), in about 93 000 individuals registered with about 100 primary care physicians in 9 general practices in Oxfordshire, UK.^4–6^ CUHK-SR is an ongoing stroke registry at Prince of Wales Hospital, a regional hospital in Hong Kong with a comprehensive stroke service team; all acute stroke or TIA patients (predominantly Chinese) in the catchment area requiring inpatient or outpatient treatment were registered in the stroke registry.

Intracranial vascular imaging was done routinely in all patients in OXVASC from April 2010 onwards. We attempted to obtain as high an imaging rate as possible by using MRA as first choice, CTA if MRI was contraindicated, and transcranial Doppler (TCD) if CTA was also contraindicated.^5 6^ In CUHK-SR, MRA was also the first-choice imaging modality, CTA was used if MRI was contraindicated or to confirm MRA findings; Occasionally, patients had both brain MRI and CT exams for clinical purposes; TCD was conducted in most patients especially when MRA and CTA were both contraindicated or unavailable.

In both studies, we collected demographic data, history of smoking, hypertension, diabetes, dyslipidaemia, ischaemic stroke, TIA and ischaemic heart disease, history of atrial fibrillation (AF) and newly diagnosed AF after an index TIA/stroke, and stroke severity by NIHSS. History of hypertension, diabetes or dyslipidaemia was defined as being previously diagnosed or taking relevant medications at the index stroke/TIA.

### Imaging protocol and assessment of ICAS

MRI exam included T1/T2-weighted imaging, diffusion-weighted imaging and time-of-flight MRA. MRI exams for OXVASC patients were conducted with a Verio V.3.0 Tesla scanner (Siemens, Germany) at Advanced Vascular Imaging Centre, University of Oxford; and for CUHK-SR patients Achieva V.3.0 Tesla scanner (Philips, Netherlands) at Prince of Wales Hospital, Hong Kong. Brain CT and single-phase CTA were performed with a 64-slice CT scanner (Toshiba Aquilion 64) at John Radcliffe Hospital, Oxford, for OXVASC patients; or a 64-slice CT scanner (Lightspeed VCT, GE Healthcare, USA) at Prince of Wales Hospital, Hong Kong, for CUHK-SR patients.

Presence of ICAS was defined as ≥50% stenosis or occlusion in any of the 11 major intracranial arteries in MRA/CTA: bilateral intracranial internal carotid arteries (ICA), middle cerebral arteries (MCA, M1 and M2), anterior cerebral arteries (ACA, A1 and A2), posterior cerebral arteries (PCA, P1 and P2), vertebral arteries (VA, V4) and basilar artery (BA). The percentage of stenosis was defined by the Warfarin-Aspirin Symptomatic Intracranial Disease method, which was the percent reduction in vessel diameter at the stenotic throat comparing with a proximal normal vessel diameter.^7^ We attempted to exclude cases with ICAS due to definite Moyamoya disease, arterial dissection, or vasculitis. We assessed interobserver agreement for presence of ICAS in 60 patients randomly selected from each study: OXVASC (XL and RH) and CUHK-SR (XL and XF).

### Statistical analyses

We compared patients’ characteristics between the two cohorts, and characteristics of those with and without ICAS in each cohort. We compared ICAS prevalence between the two cohorts in different age categories (<60, 60–69, 70–79 and ≥80 years), and in patients with 0, 1, 2 or 3 vascular risk factors (histories of hypertension, diabetes and dyslipidaemia). We compared prevalence of common vascular risk factors between the two cohorts in different age categories. We also compared distribution of ICAS lesions (in anterior/posterior circulations and in individual arteries) between the two cohorts. Means (SD) or medians (IQR) were used for describing continuous variables and numbers (percentage) for categorical variables. Student’s t-tests were used for comparison of continuous variables between two groups and χ^2^ tests for categorical variables. Interobserver agreement for presence of ICAS was assessed with Cohen’s kappa.

Univariate and multivariate logistic regression analyses were conducted for risk factors of ICAS in each cohort; crude and adjusted ORs and the 95% CI were presented. We also conducted subgroup analyses for independent risk factors of ICAS in patients aged< or ≥70 years in each cohort. Moreover, the difference between the two cohorts in ICAS prevalence was presented with crude, age-adjusted and multivariable-adjusted ORs (95% CI), among all patients, and in those aged< or ≥70 years; p for study centre–age (<or ≥70 years) interaction was obtained. All statistical analyses were conducted using SPSS Statistics V.22.0 (IBM). Two-tailed p<0.05 was considered statistically significant.

### Systematic review of ICAS prevalence in TIA/ischaemic stroke patients

The systematic review was carried out according to the Meta-analysis Of Observational Studies in Epidemiology^8^ and the Preferred Reporting Items for Systematic Reviews and Meta-Analyses^9^ statements. We searched PubMed and OVID on 14 October 2019, for primary studies reporting ICAS prevalence in TIA and/or ischaemic stroke patients, with full-text article published in English since 1 January 1990. Briefly, the search terms included ICAS, ischaemic stroke, TIA and prevalence (search strategy provided in online supplemental etables 1 & 2). We also manually searched references in pertinent review articles.

10.1136/jnnp-2020-325630.supp1 Supplementary data

The inclusion criteria were studies reporting prevalence of ICAS (> or ≥50% stenosis or occlusion) in TIA and/or ischaemic stroke patients with a sample size ≥100. Studies were excluded if it reported ICAS prevalence in highly selective (eg, non-cardioembolic) stroke patients; prevalence of complete occlusion (rather than stenosis and occlusion) of intracranial arteries; or ICAS in certain (eg, anterior-circulation arteries only) rather than all major intracranial arteries. The study quality was assessed with a modified version of the Newcastle-Ottawa Scale, with a total score of 0–5 and score of ≥3 or <3, respectively, indicating low and high risk of bias (more details provided in the Appendix).^10 11^ We collected the following information from relevant studies: country/region, enrolment period, sample size, mean age, male percentage, TIA and/or ischaemic stroke patients involved, imaging modality and criteria to define ICAS, and prevalence of any ICAS and/or symptomatic ICAS.

### Data availability statement

Data are available on reasonable request. All data relevant to the study are included in the article or uploaded as (online supplemental information). Requests for access to anonymised data reported in this paper will be considered by the corresponding author.

## Results

Patient recruitment and investigation in the two studies are shown in (online supplemental efigure 1). Of 1579 potentially eligible patients in OXVASC, 1368 (86.6%) underwent intracranial vascular imaging (1033/65.4% MRA; 254/16.1% CTA; 81/5.1% TCD only), whereas 154 (9.8%) had only carotid ultrasound imaging (often due to contraindications to MRA and CTA) and 57 (3.6%) did not undergo any vascular imaging. Of 1099 potentially eligible patients in CUHK-SR, 953 (86.7%) underwent intracranial vascular imaging (673/61.2% MRA; 99/9.0% CTA; 81/7.4% MRA and CTA; 262/23.8% TCD only; 1/0.1% digital subtraction angiography (DSA) only), 38 (3.5%) had only carotid ultrasound imaging and 107 (9.7%) did not undergo any vascular imaging (details in (online supplemental etable 3). In both cohorts, patients who did not receive intracranial MRA/CTA were older with a higher burden of vascular risk factors (details in (online supplemental etable 4).

Among the 1287 TIA and minor stroke patients in OXVASC and 691 in CUHK-SR with brain MRA and/or CTA, CUHK-SR patients were younger (66 vs 69 years old; p<0.001) and more of them were male (63.8% vs 51.8%; p<0.001). More OXVASC patients had TIA as an index ischaemic event (65.4% vs 30.1%; p<0.001). More CUHK-SR patients had histories of hypertension, diabetes, dyslipidaemia and previous ischaemic stroke or TIA, while more OXVASC patients had history of ischaemic heart disease, and more of them had AF (table 1). Additionally, more male patients in OXVASC had ever smoked than male patients in CUHK-SR (64.7% vs 39.5%; p<0.001), irrespective of age, while the ever-smoking rates were similar between the two cohorts in females (online supplemental efigure 2). When classified to four age groups, more CUHK-SR patients had histories of hypertension, diabetes and dyslipidaemia than OXVASC patients in each age group (all p<0.05;online supplemental efigure 2).

**Table 1:** Baseline characteristics of patients in OXVASC and CUHK-SR

Characteristics	OXVASC (n=1287)	CUHK-SR (n=691)	P value
Age, years	69 (13.9)	66 (12.3)	<0.001
Age ≥70 years	715 (55.6)	279 (40.4)	<0.001
Male sex	667 (51.8)	441 (63.8)	<0.001
Ever-smoker	688 (53.5)	270 (39.1)	<0.001
Male smoker	431 (64.7)	174 (39.5)	<0.001
Female smoker	257 (41.5)	96 (38.4)	0.407
History of hypertension	707 (54.9)	454 (65.6)	<0.001
History of diabetes	169 (13.1)	181 (26.2)	<0.001
History of dyslipidaemia	436 (33.9)	354 (51.2)	<0.001
Atrial fibrillation*	191 (14.9)	78 (11.3)	0.028
History of ischaemic stroke or TIA	192 (14.9)	144 (20.8)	0.001
History of ischaemic heart disease	166 (12.9)	54 (7.8)	<0.001
Event type			<0.001
TIA	842 (65.4)	208 (30.1)	
Minor stroke	445 (34.6)	483 (69.9)	
Presence of ICAS	257 (20.0)	297 (43.0)	<0.001
No of arteries† affected			<0.001
0	1030 (80.0)	394 (57.0)	
1	162 (12.6)	148 (21.4)	
≥2	95 (7.4)	149 (21.6)	

Values are means (SD) or medians (IQR) or numbers (%); Student’s t-tests were used for comparison of continuous variables and χ^2^ tests for categorical variables.

*History of atrial fibrillation and newly diagnosed atrial fibrillation after the index stroke or TIA.

†Including 11 cerebral arteries: bilateral intracranial internal carotid arteries, middle/anterior/posterior cerebral arteries, intracranial vertebral arteries and basilar artery.

CUHK-SR, the Chinese University of Hong Kong Stroke Registry; ICAS, intracranial atherosclerotic stenosis; OXVASC, Oxford Vascular Study; TIA, transient ischaemic attack.

Interobserver agreement for presence of ICAS was good in both cohorts (both kappa=0.82). ICAS prevalence was higher in Chinese (CUHK-SR) than in Caucasians (OXVASC): 43.0% vs 20.0% (OR 3.02; 95% CI 2.47 to 3.70; p<0.001). More Chinese patients had ≥2 cerebral arteries with ICAS than Caucasians (21.6% vs 7.4%; p<0.001; table 1).

**Figure 1:**
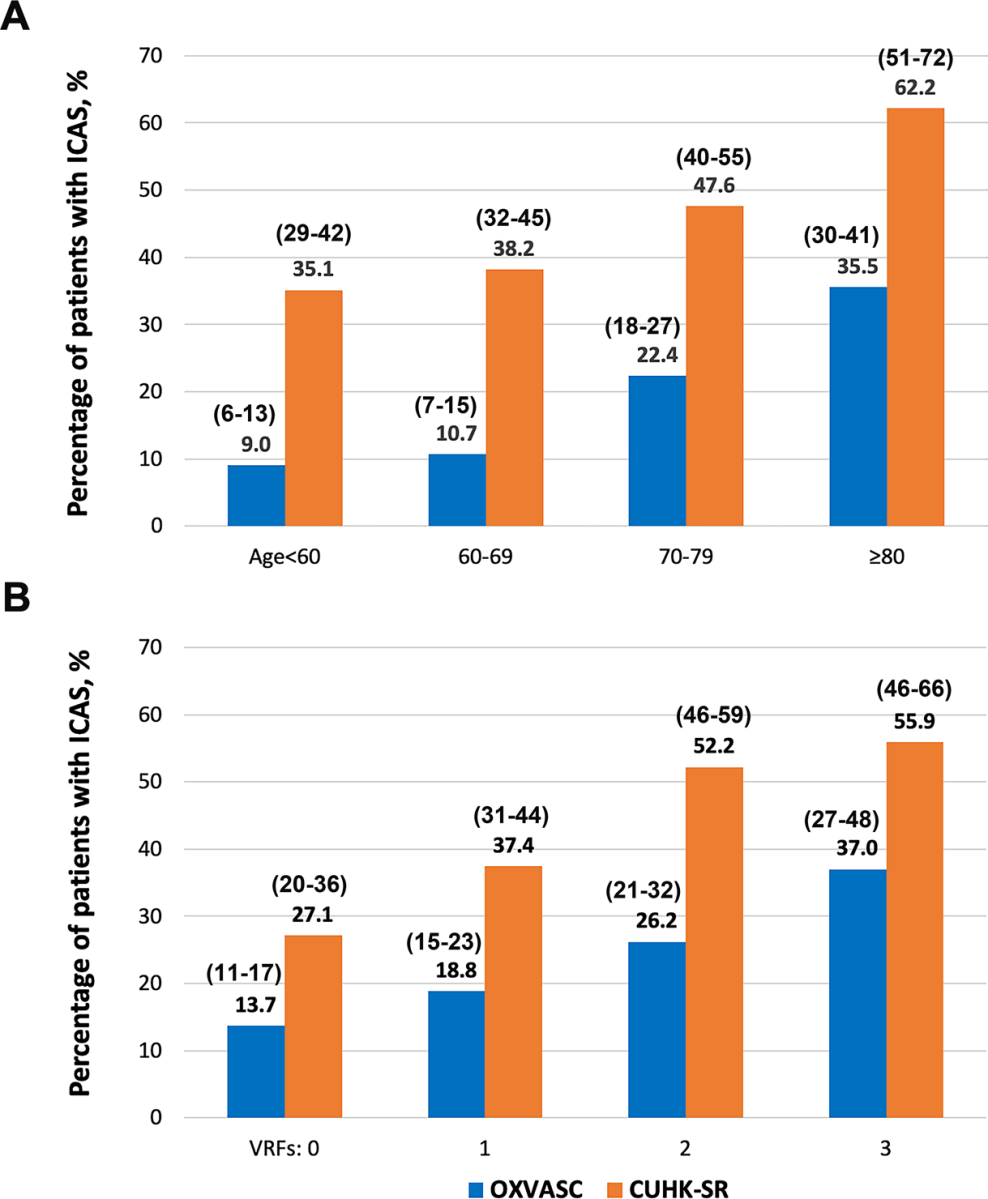
Prevalence of ICAS in minor stroke and TIA patients in Caucasians (OXVASC) and Chinese (CUHK-SR) in subgroups. (A) Prevalence of ICAS in OXVASC and CUHK-SR in different age groups; p<0.001 for χ^2^ tests in the comparison between OXVASC and CUHK-SR in each age group. Larger differences in ICAS prevalence are seen in younger patients. (B) Prevalence of ICAS in OXVASC and CUHK-SR by different numbers (0, 1, 2 or 3) of vascular risk factors including histories of hypertension, diabetes and dyslipidaemia; p<0.001 for χ^2^ tests in the comparison between OXVASC and CUHK-SR in subgroups of patients with 0, 1 or 2 vascular risk factors, and p=0.008 in the subgroup of patients with three vascular risk factors.95% CIs of ICAS prevalence in these subgroups are provided within parentheses in the figure. CUHK-SR, the Chinese University of Hong Kong stroke Registry; ICAS, intracranial atherosclerotic stenosis; OXVASC, Oxford vascular study; TIA, transient ischaemic attack; VRFs, number of vascular risk factors.

Chinese patients with ICAS were younger than Caucasians (68 vs 75 years; p<0.001). The ethnic difference in ICAS prevalence was independent of age (age-adjusted OR 3.73; 95% CI 3.00 to 4.63; p<0.001), which existed in those aged <60, 60–69, 70–79 and ≥80 years (figure 1A). However, the difference was greater (p-interaction=0.005) at age <70 years (36.7% vs 9.8%; OR 5.33; 95% CI 3.79 to 7.50; p<0.001) than at ≥70 years (52.3% vs 28.1%; OR 2.81; 95% CI 2.11 to 3.74; p<0.001). Only in Caucasians aged ≥80 did the prevalence of ICAS (35.5%; 95% CI 30% to 41%) reach that of Chinese patients aged <60 years (35.1%; 95% CI 29% to 42%).

Table 2 shows multiple factors associated with presence of ICAS in both cohorts, including older age, histories of hypertension, diabetes, dyslipidaemia and ischaemic stroke or TIA; while AF and history of ischaemic heart disease were associated with ICAS in OXVASC but not CUHK-SR. In addition, more patients with ICAS had minor stroke as the index ischaemic event than those without ICAS in OXVASC (39.7% vs 33.3%; p=0.054); but the ischaemic event types were similar between those with or without ICAS in CUHK-SR.

**Table 2:** Baseline characteristics of patients with or without ICAS in OXVASC and CUHK-SR

Characteristics	OXVASC (n=1287)			CUHK-SR (n=691)		
	Any ICAS (n=257)	No ICAS (n=1030)	P value	Any ICAS (n=297)	No ICAS (n=394)	P value
Age, years	75 (12.2)	68 (13.8)	<0.001	68 (12.3)	64 (11.9)	<0.001
Age ≥70 years	201 (78.2)	514 (49.9)	<0.001	146 (49.2)	133 (33.8)	<0.001
Male sex	141 (54.9)	526 (51.1)	0.276	184 (62.0)	257 (65.2)	0.375
Ever-smoker	138 (53.7)	550 (53.4)	0.943	116 (39.1)	154 (39.1)	0.994
Male smoker	90 (63.8)	341 (65.0)	0.804	76 (41.3)	98 (38.1)	0.502
Female smoker	48 (41.4)	209 (41.5)	0.986	40 (35.4)	56 (40.9)	0.375
History of hypertension	180 (70.0)	527 (51.2)	<0.001	219 (73.7)	234 (59.4)	<0.001
History of diabetes	49 (19.1)	120 (11.7)	0.002	101 (34.0)	80 (20.3)	<0.001
History of dyslipidaemia	111 (43.2)	325 (31.6)	<0.001	173 (58.2)	181 (45.9)	0.001
Atrial fibrillation*	59 (23.0)	132 (12.8)	<0.001	30 (10.1)	48 (12.2)	0.392
History of ischaemic stroke or TIA	59 (23.0)	133 (12.9)	<0.001	77 (25.9)	67 (17.0)	0.004
History of ischaemic heart disease	59 (23.0)	107 (10.4)	<0.001	28 (9.4)	26 (6.6)	0.170
Event type			0.054			0.814
TIA	155 (60.3)	687 (66.7)		88 (29.6)	120 (30.5)	
Minor stroke	102 (39.7)	343 (33.3)		209 (70.4)	274 (69.5)	

Values are mean (SD) or median (IQR) or number (%); Student’s t-tests were used for comparison of continuous variables and χ^2^ tests for categorical variables.

*History of atrial fibrillation and newly diagnosed atrial fibrillation after the index stroke or TIA.

CUHK-SR, the Chinese University of Hong Kong Stroke Registry; ICAS, intracranial atherosclerotic stenosis; OXVASC, Oxford Vascular Study; TIA, transient ischaemic attack.

After adjusting for age, histories of hypertension, diabetes and dyslipidaemia were significantly associated with ICAS in both cohorts, while male sex was significantly associated with ICAS in OXVASC only. Multivariate logistic regression identified older age, and histories of hypertension and diabetes as independent risk factors of ICAS in both cohorts; male sex was an independent risk factor of ICAS in OXVASC; history of dyslipidaemia tended to be independently associated with ICAS in CUHK-SR (table 3).

**Table 3:** Risk factors for presence of ICAS in the two cohorts

Risk factors	OXVASC (n=1287)						CUHK-SR (n=691)					
	Crude OR (95% CI)	P value	Age-adjusted OR (95% CI)	P value	Multivariable-adjusted OR (95% CI)	P value	Crude OR (95% CI)	P value	Age-adjusted OR (95% CI)	P value	Multivariable-adjusted OR (95% CI)	P value
Age (every 10 years increment)	1.65 (1.46 to 1.87)	<0.001	–	–	1.62 (1.42 to 1.84)	<0.001	1.33 (1.17 to 1.52)	<0.001	–	–	1.23 (1.08 to 1.41)	0.003
Male sex	1.17 (0.89 to 1.53)	0.276	1.40 (1.05 to 1.86)	0.022	1.36 (1.01 to 1.84)	0.042	0.87 (0.64 to 1.19)	0.375	0.91 (0.66 to 1.25)	0.555	0.96 (0.70 to 1.33)	0.810
Ever-smoker	1.01 (0.77 to 1.33)	0.943	1.12 (0.84 to 1.48)	0.445	0.98 (0.73 to 1.32)	0.910	1.00 (0.73 to 1.36)	0.994	1.01 (0.74 to 1.38)	0.967	1.02 (0.74 to 1.40)	0.894
History of hypertension	2.23 (1.66 to 2.99)	<0.001	1.67 (1.23 to 2.27)	0.001	1.51 (1.09 to 2.08)	0.012	1.92 (1.38 to 2.66)	<0.001	1.64 (1.17 to 2.31)	0.004	1.43 (1.00 to 2.03)	0.047
History of diabetes	1.79 (1.24 to 2.57)	0.002	1.74 (1.19 to 2.53)	0.004	1.46 (0.98 to 2.16)	0.057	2.02 (1.44 to 2.85)	<0.001	1.86 (1.32 to 2.64)	<0.001	1.67 (1.17 to 2.38)	0.005
History of dyslipidaemia	1.65 (1.25 to 2.18)	<0.001	1.42 (1.06 to 1.89)	0.017	1.17 (0.86 to 1.58)	0.319	1.64 (1.21 to 2.23)	0.001	1.50 (1.10 to 2.05)	0.010	1.34 (0.98 to 1.85)	0.069

CUHK-SR, the Chinese University of Hong Kong Stroke Registry; ICAS, intracranial atherosclerotic stenosis; OXVASC, Oxford Vascular Study.

Subgroups analyses (online supplemental etable 5) showed that in patients aged ≥70 years, older age was an independent risk factor of ICAS in both cohorts; in addition, history of hypertension and history of diabetes were respectively independent risk factors of ICAS in OXVASC and CUHK-SR. In those younger than 70 years, male sex (p=0.079) and history of hypertension (p=0.090), and history of diabetes (p=0.054) tended to be, respectively, independently associated with ICAS in OXVASC and CUHK-SR.

ICAS prevalence was equivalent in Chinese patients with no vascular risk factors (27.1%; 95% CI 20% to 36%) and Caucasians with two vascular risk factors (26.2%; 95% CI 21% to 32%; figure 1B). The higher ICAS prevalence in Chinese than Caucasians was independent of vascular risk factors (multivariable-adjusted OR 3.21; 95% CI 2.56 to 4.02; p<0.001), which existed in subgroups of patients with 0–3 vascular risk factors (figure 1B).

ICAS locations were also different between the two cohorts. The anterior and posterior circulations (both 12.0%) were similarly involved with ICAS in Caucasian patients, while Chinese patients had more ICAS lesions in the anterior than posterior circulation (35.9 vs 17.6%). Overall, more Chinese patients than Caucasians had ICAS lesions in either or both circulations (all p<0.05; table 4).

**Table 4:** Distribution of ICAS lesions in the two cohorts

	OXVASC (n=1287)		CUHK-SR (n=691)	
	No of patients with the arteries affected/no of patients with the artery imaged (%)	No of arteries affected/total no of arteries imaged (%)	No of patients with the arteries affected/ no of patients with the artery imaged (%)	No of arteries affected/total no of arteries imaged (%)
Any ICAS in anterior circulation	155/1287 (12.0)*		248/691 (35.9)*	
Any ICAS in posterior circulation	155/1287 (12.0)†		121/689 (17.6)†	
ICAS in both anterior and posterior circulations	53/1287 (4.1)‡		72/689 (10.4)‡	
ICAS in individual arteries				
Intracranial ICA	68/1287 (5.3)	90/2574 (3.5)	83/691 (12.0)	107/1382 (7.7)
ACA (A1+A2)	30/1287 (2.3)	31/2574 (1.2)	68/691 (9.8)	75/1382 (5.4)
MCA (M1+M2)	85/1287 (6.6)	93/2574 (3.6)	184/691 (26.6)	228/1382 (16.5)
PCA (P1+P2)	83/1287 (6.4)	98/2574 (3.8)	102/691 (14.8)	116/1382 (8.4)
VA (V4)	82/1287 (6.4)	97/2574 (3.8)	15/334 (4.5)	19/668 (2.8)
BA	13/1287 (1.0)	13/1287 (1.0)	23/689 (3.3)	23/689 (3.3)

*P<0.001 for comparison between the two cohorts.

†P=0.001 for comparison between the two cohorts.

‡P<0.001 for comparison between the two cohorts.

ACA, anterior cerebral artery; BA, basilar artery; CUHK-SR, the Chinese University of Hong Kong Stroke Registry; ICA, internal carotid artery; ICAS, intracranial atherosclerotic stenosis; MCA, medial cerebral artery; OXVASC, Oxford Vascular Study; PCA, posterior cerebral artery; VA, vertebral artery.

In OXVASC, PCA (percentage of arteries with ICAS, 3.8%), VA-V4 (3.8%), MCA (3.6%) and intracranial ICA (3.5%) were similarly affected by ICAS, while ACA (1.2%) and BA (1.0%) were less frequently affected (table 4). In CUHK-SR, the most commonly affected cerebral artery was MCA (16.5%), followed by PCA (8.4%), intracranial ICA (7.7%), ACA (5.4%), BA (3.3%) and VA-V4 (2.8%). Per-patient and per-artery data of ICAS locations are presented in table 4.

### Systematic review of ICAS prevalence in TIA/ischaemic stroke patients

Of the 889 records retrieved from literature search, we identified 32 studies reporting ICAS prevalence in TIA and/or ischaemic stroke patients in various populations published in the last three decades (online supplemental efigure 3). Studies conducted in different continents are, respectively, summarised in (online supplemental etables 6–9). Different methods/criteria were used to define ICAS: various velocity criteria by TCD, and/or arterial luminal narrowing by MRA/CTA/DSA. Among the four European studies with over 1000 patients (mostly Caucasians),^12–15^ ICAS was diagnosed by TCD alone in most or all patients,^14 15^ or screened by TCD and confirmed with MRA/CTA/DSA only in those with suspected ICAS in TCD (online supplemental etable 6).^12 13^ In large-scale studies in Asia, MRA or MRA/CTA were used in most or all patients (online supplemental etable 7).^16–18^ In the worldwide TIAregistry.org project,^19^ 67%, 46% and 15% of the patients had TCD, MRA and CTA, respectively, when ICAS was diagnosed with one or more of these imaging modalities (online supplemental etable 9). Among the 32 studies included, 26 (81.3%) had a modified Newcastle-Ottawa Scale of 3 or 4, indicating a low risk of bias (online supplemental etable 6–9).

Overall, the prevalence of any ICAS or symptomatic ICAS was higher in Asian than European patients: 22%–65% vs 7%–36% and 17%–36% vs 0.5%–31%, respectively (online supplemental etables 6 and 7). Studies in the USA mostly involved patients with multiple ethnic backgrounds (online supplemental etables 8). There was no direct comparison between Caucasian and Asian (or Chinese) TIA/stroke patients in ICAS prevalence in any individual study. Although higher ICAS prevalence in 345 Japanese TIA/minor stroke patients than 4238 non-Japanese patients (20 vs 13%; p=0.01) was reported in the TIAregistry.org cohort, there were at least 912 patients of Asian, African or Hispanic ancestry in the non-Japanese patients; moreover, no data were reported in this study for the locations and risk factors of ICAS in different ethnic groups.^20^

## Discussion

To our knowledge based on the systematic review, the current study was the first to directly compare the prevalence, distribution and risk factors of ICAS between Caucasian and Chinese patients with TIA/minor stroke, based on two contemporaneous cohorts (1287 patients from Oxford and 691 from Hong Kong), with similar imaging modalities (MRA/CTA) and criteria to define ICAS. Overall, ICAS prevalence was higher among Chinese TIA/minor stroke patients than Caucasians (43.0% vs 20.0%; p<0.001), and Chinese patients with ICAS were younger than Caucasians. This ethnic difference in ICAS prevalence was independent of age and vascular risk factors, but was most marked at younger ages. ICAS prevalence was equivalent in Chinese aged <60 years and Caucasians aged ≥80, and in Chinese with no vascular risk factors and Caucasians with two vascular risk factors.

The higher ICAS prevalence and younger age of patients with ICAS in Chinese than Caucasian patients in the current study corroborated the higher susceptibility of Chinese to ICAS than Caucasians. Interestingly, the independent associations between vascular risk factors and presence of ICAS was weaker in patients aged <70 years than ≥70 years in the Chinese cohort. Therefore, the higher prevalence of vascular risk factors in Chinese patients cannot explain all of the ethnic difference in ICAS prevalence, especially in younger patients.

Previous studies in Asia indicated stronger association of atherosclerosis in extracranial and coronary arteries, than that in intracranial and coronary arteries, independent of vascular risk factors, among subjects with or without ischaemic stroke.^21–24^ Therefore, we speculated that atherosclerosis of intracranial arteries might be a relatively independent and earlier process in systemic atherosclerosis in Asians.^24^ In addition to the effects of modifiable vascular risk factors (eg, hypertension, diabetes and dyslipidaemia), genetic factors play an important role in early ICAS development in Asians.^25^

Large-scale genome-wide association studies and subsequent meta-analyses have revealed genetic variants associated with large artery atherosclerotic (LAA) stroke.^26 27^ For instance, MEGASTROKE, the most valuable multiancestry genome-wide association study in stroke patients (with 40 585 and 17 369 stroke cases, respectively, in the European and East Asian cohorts) have indicated some genetic loci associated with LAA stroke shared across continents by transancestral meta-analysis, while there are also genetic loci associated with LAA stroke in European cohorts only.^27^ These findings have implied genetic differences underlying LAA stroke among populations. However, atherosclerotic disease of extracranial and intracranial arteries was not differentiated in such analyses, and mostly, only symptomatic (rather than ‘any’) large artery disease was studied.^26 27^ Thus far, evidence is insufficient regarding the genetic background of higher ICAS prevalence in Asians than in Caucasians. Some Korean and Japanese studies showed that over 20% of patients with non-Moyamoya intracranial stenosis had ring finger protein 213 gene variants (particularly the p.R4810K variant), which could lead to vascular fragility.^28^ The p.R4810K variant is commonly seen in East Asian populations but rarely seen in Caucasians.^29–31^ This may be a contributing factor for the higher ICAS prevalence in Asians than Caucasians. In addition, geographical and environmental factors, such as the climate, food, social and cultural habits, may also underlie the ethnic differences in ICAS prevalence. There is indeed geographic difference between North (higher) and South China in ICAS prevalence among ischaemic stroke and TIA patients.^32^

Although lower than that in Chinese, ICAS prevalence in Caucasians was higher than previously estimated in European studies of TIA/ischaemic stroke patients (online supplemental etable 6), but this will partly reflect the reliance on TCD only in many previous studies. In the current study, ICAS presented in 22.4% of Caucasians aged 70–79 years and 35.5% aged ≥80 (figure 1). Intracranial vascular imaging might, therefore, be justified in elderly Caucasian patients presenting with TIA/stroke with multiple vascular risk factors, for better understanding of the stroke aetiology.

The current study indicated more involvement of the anterior circulation by ICAS in Chinese stroke/TIA patients, with MCA as the most common lesion location, consistent with previous Chinese studies.^33–35^ For Caucasian patients, our study indicated same involvement rates of the anterior and posterior circulations by ICAS, with PCA, VA-V4, MCA and ICA similarly affected. Distribution of ICAS lesions in anterior vs posterior circulations in European Caucasians varied in previous studies. A study in Netherlands (n=786) reported higher ICAS prevalence in posterior circulation in TIA/ischaemic stroke patients, with PCA and distal VA most commonly affected,^36^ while other European studies reported higher ICAS prevalence in the anterior circulation.^13 14 37^ Previous American (multiple ethnic backgrounds) and Chinese studies suggested that atherosclerosis might affect arteries in the anterior circulation earlier in life, and vascular risk factors and genetic factors might interface differently with endothelium of cerebral arteries with different haemodynamic and structural features.^35 38^ Yet, these inferences need further investigations.

Our study had limitations. First, although we attempted to exclude non-atherosclerotic stenosis of intracranial arteries, we used MRA/CTA to define ICAS, which might not accurately differentiate the etiologies of ICAS; even with DSA, the currently gold standard to define ICAS, it is difficult to differentiate ICAS and early-stage Moyamoya disease. Therefore, there might be contamination from non-atherosclerotic stenosis (eg, early-stage Moyamoya disease or arterial dissection) in ICAS in the current study, especially in younger Chinese patients without vascular risk factors. Moreover, to determine the degree of luminal stenosis in ICAS, time-of-flight MRA, with a flow-dependent nature, tends to overestimate the severity, while good-quality CTA is more precise.^39 40^ Second, stroke subtyping and symptomatic/asymptomatic ICAS were prospectively determined as soon as possible after patient recruitment in OXVASC,^6^ while retrospective classification was needed in a considerable proportion of TIA patients in CUHK-SR with atypical neurological symptoms without cerebral infarctions, or those with multiple possible stroke etiologies, which might be unreliable. We, therefore, reported the prevalence of any ICAS but did not differentiate symptomatic and asymptomatic ICAS, or the stroke aetiologies, in the current study. Moreover, a considerable proportion of patients did not have complete V4 scanned in MRA/CTA in CUHK-SR, who were not counted in the analyses of ICAS locations; thus, the prevalence of V4 stenosis in Chinese patients may need further verification. Finally, data were not available for extracranial carotid and vertebral artery stenosis for the Chinese patients, hence we did not compare prevalence of extracranial arterial stenosis between the two cohorts; also, without considering the extracranial artery status, accuracy in grading the degree of intracranial stenosis as well as determining the compensatory effects mimicking an intracranial stenosis in the presence of extracranial occlusion might have been underestimated or misinterpreted. Future longitudinal studies with both extracranial- and intracranial vascular imaging, and with a more comprehensive profile of patients’ characteristics (eg, peripheral artery disease, chronic kidney disease, medications and vascular risk factor management status) and environmental factors (eg, climate, food, social and cultural habits), will better delineate and explain the ethnic difference in cervicocerebral arterial stenosis between the two populations.

In conclusion, among TIA and minor stroke patients, Chinese are more susceptible to ICAS than Caucasians, with an earlier onset age and a higher prevalence, independent of vascular risk factors. ICAS prevalence was equivalent in Chinese aged <60 years and Caucasians aged ≥80. There are also significant differences between Chinese and Caucasians in ICAS locations. Overall, ICAS shared similar risk factors in Chinese and Caucasian patients, but vascular risk factors were not independently associated with ICAS in Chinese patients aged <70 years, supporting an important role of genetic factors underlying the ethnic difference. On the other hand, ICAS burden in Caucasians might be higher than previously estimated, especially in the elderly.

## Data Availability

Data are available on reasonable request. All data relevant to the study are included in the article or uploaded as online supplemental information. Requests for access to anonymised data reported in this paper will be considered by the corresponding author.
