# Supplementary material for: Intracranial arterial stenosis in Caucasian versus Chinese patients with TIA and minor stroke: two contemporaneous cohorts and a systematic review
Source: J Neurol Neurosurg Psychiatry. 2021 Mar 30;92(6):590–7. doi: 10.1136/jnnp-2020-325630 (PMC8142447; doi:10.1136/jnnp-2020-325630)
Supplement: Supplementary data [file jnnp-2020-325630supp001.pdf]

Leng et al, Intracranial arterial stenosis in Caucasian versus Chinese patients with TIA and minor stroke: two contemporaneous cohorts and a systematic review

Supplemental materials

eTable 1. Search strategy for PubMed, conducted on 14 October 2019 2

eTable 2. Search strategy for OVID, conducted on 14 October 2019 3

eTable 3: Details of imaging received by study patients 4

eTable 4: Baseline characteristics of study population, stratified by intracranial vascular imaging 5

eTable 5. Risk factors of ICAS in younger and older patients in the 2 cohorts 6

eTable 6. Prevalence of ICAS in patients with ischemic stroke or TIA in Europe 7

eTable 7. Prevalence of ICAS in patients with ischemic stroke or TIA in Asia 9

eTable 8. Prevalence of ICAS in patients with ischemic stroke or TIA in North and South America 11

eTable 9. Prevalence of ICAS in patients with ischemic stroke or TIA in other countries/regions or worldwide 12

eFigure 1. Flow chart for patient screening in the current study 13

eFigure 2. Age-specified prevalence of vascular risk factors in the 2 cohorts 14

eFigure 3. Flow chart for study screening and selection in the systematic review 15

Appendix. Modified Newcastle-Ottawa Scale (NOS) for quality assessment of the primary studies 16

References in the supplemental materials 17

Leng X, *et al. J Neurol Neurosurg Psychiatry* 2021; 92:590–597. doi: 10.1136/jnnp-2020-325630

eTable 1. Search strategy for PubMed, conducted on 14 October 2019

Search #	Search queries	Number of items found
#1	Search (intracranial arter* steno* OR intracranial steno* OR intracranial large artery steno* OR intracranial occlusi* OR intracranial arter* occlusi* OR intracranial large artery occlusi* OR intracranial atherosclero* OR intracranial large artery atherosclero*) [All Fields]	2,388
#2	Search (prevalence OR incidence OR frequency OR epidemiology OR burden) [All Fields]	3,831,047
#3	Search (ischemic stroke OR ischaemic stroke OR minor stroke OR minor ischemic stroke OR minor ischaemic stroke OR transient ischemic attack OR transient ischaemic attack OR TIA) [All Fields]	113,316
#4	Search #1 AND #2 AND #3	373
#5	Search #4 AND ("1990/01/01" [Date - Publication]: "2019/10/14" [Date - Publication]) AND English [Language]	324

Leng X, *et al. J Neurol Neurosurg Psychiatry* 2021; 92:590–597. doi: 10.1136/jnnp-2020-325630

eTable 2. Search strategy for OVID, conducted on 14 October 2019 [a]

Search #	Search queries	Number of items found
#1	(intracranial arter* steno* or intracranial steno* or intracranial large artery steno* or intracranial occlusi* or intracranial arter* occlusi* or intracranial large artery occlusi* or intracranial atheroscleró* or intracranial large artery atherosclero*).mp. [mp=ti, ab, hw, tn, ot, dm, mf, dv, kw, fx, dq, nm, kf, ox, px, rx, an, ui, sy]	8,452
#2	(prevalence or incidence or frequency or epidemiology or burden).mp. [mp=ti, ab, hw, tn, ot, dm, mf, dv, kw, fx, dq, nm, kf, ox, px, rx, an, ui, sy]	10,186,862
#3	(ischemic stroke or ischaemic stroke or minor stroke or minor ischemic stroke or minor ischaemic stroke or transient ischemic attack or transient ischaemic attack or TIA).mp. [mp=ti, ab, hw, tn, ot, dm, mf, dv, kw, fx, dq, nm, kf, ox, px, rx, an, ui, sy]	231,419
#4	#1 AND #2 AND #3	1293
#5	limit #4 to yr="1990 -Current"	1283
#6	limit #5 to (English) AND (full text) AND (human)	563

[a] The following databases were searched via OVID: Embase 1910 to Present; Ovid MEDLINE(R) 1946 to October 14, 2019; and Ovid MEDLINE(R) and Epub Ahead of Print, In-Process & Other Non-Indexed Citations 1946 to October 14, 2019.

eTable 3: Details of imaging received by study patients

Imaging modality	OXVASC<br>n (%)<br>N= 1579	CUHK-SR<br>n (%)<br>N= 1099
Intracranial vascular imaging		
MR angiography	1035 (65.5)	673 (61.2)
CT angiography	253 (16.0)	99 (9.0)
MR and CT angiography	0	81 (7.4)
Transcranial Doppler only	81 (5.2)	262 (23.8)
Digital subtraction angiography only	0	1 (0.1)
Extracranial vascular imaging only		
Carotid Doppler ultrasound	154 (9.8)	38 (3.5)
No vascular imaging	57 (3.6)	107 (9.7)
Known atrial fibrillation	25	62
Previously investigated or imaged elsewhere	8	0
Other clinical or logistical issue [a]	24	45

Abbreviations: OXVASC, Oxford Vascular Study; CUHK-SR, the Chinese University of Hong Kong Stroke Registry.

[a] Such as older than 90 years, gastrointestinal bleeding, trauma, dementia.

Leng X, *et al. J Neurol Neurosurg Psychiatry* 2021; 92:590–597. doi: 10.1136/jnnp-2020-325630

BMJ Publishing Group Limited (BMJ) disclaims all liability and responsibility arising from any reliance placed on this supplemental material which has been supplied by the author(s)

eTable 4: Baseline characteristics of study population, stratified by intracranial vascular imaging.

Characteristics	OXVASC			CUHK-SR		
	Intracranial MRA/CTA (n= 1,287)	***No intracranial MRA/CTA (n= 292)***	**p-value**	***Intracranial MRA/CTA (n= 691)***	***No intracranial MRA/CTA (n= 408)***	**p-value**
Age, years	69 (13.9)	75 (12.8)	<0.0001	66 (12.3)	72 (11.9)	<0.001
Age ≥70 years	715 (55.6)	209 (71.6)	<0.0001	279 (40.4)	243 (59.6)	<0.001
Male sex	667 (51.8)	143 (49.0)	0.38	441 (63.8)	213 (52.2)	<0.001
Ever-smoker	688 (53.5)	162 (56.3)	0.40	270 (39.1)	154 (37.7)	0.662
History of hypertension	707 (54.9)	194 (67.4)	<0.0001	453 (65.6)	286 (70.1)	0.121
History of diabetes	169 (13.1)	62 (21.6)	<0.0001	181 (26.2)	116 (28.4)	0.420
History of dyslipidemia	436 (33.9)	109 (38.1)	0.17	354 (51.2)	169 (41.4)	<0.002
Atrial fibrillation [a]	191 (14.9)	70 (24.3)	<0.0001	78 (11.3)	95 (23.3)	<0.001
History of ischemic stroke/TIA	192 (14.9)	88 (30.4)	<0.0001	144 (20.8)	90 (22.1)	0.633
History of ischemic heart disease	166 (12.9)	65 (22.5)	<0.0001	54 (7.8)	52 (12.7)	0.007
Event type						0.015
TIA	842 (65.4)	158 (54.1)	<0.0001	208 (30.1)	152 (37.3)	
Minor stroke	445 (34.6)	134 (45.9)		483 (69.9)	256 (62.7)	

Abbreviations: OXVASC, Oxford Vascular Study; CUHK-SR, the Chinese University of Hong Kong Stroke Registry; MRA, magnetic resonance angiography; CTA, computed tomography angiography; TIA, transient ischemic attack.

[a] History of atrial fibrillation and newly diagnosed atrial fibrillation after the index stroke or TIA.

Leng X, *et al. J Neurol Neurosurg Psychiatry* 2021; 92:590–597. doi: 10.1136/jnnp-2020-325630

eTable 5. Risk factors of ICAS in younger and older patients in the 2 cohorts

Risk factors	OXVASC (n=1287)				CUHK-SR (N=691)			
	Patients <70 years old (n=572)		Patients ≥70 years old (n=715)		Patients <70 years old (n=412)		Patients ≥70 years old (n=279)	
	Multivariable adjusted OR (95% CI)	p-value	Multivariable adjusted OR (95% CI)	p-value	Multivariable adjusted OR (95% CI)	p-value	Multivariable adjusted OR (95% CI)	p-value
Age (every 10-year increment)	0.97 (0.72-1.30)	0.818	1.79 (1.37-2.35)	<0.001	1.00 (0.78-1.28)	0.973	1.86 (1.10-3.15)	0.020
Male sex	1.72 (0.94-3.16)	0.079	1.28 (0.90-1.82)	0.173	1.01 (0.65-1.55)	0.980	1.05 (0.63-1.74)	0.858
Ever-smoker	1.01 (0.57-1.79)	0.965	0.98 (0.69-1.39)	0.909	0.94 (0.62-1.43)	0.774	1.19 (0.72-1.98)	0.494
History of hypertension	1.68 (0.92-3.06)	0.090	1.48 (1.00-2.18)	0.048	1.40 (0.90-2.20)	0.138	1.68 (0.93-3.02)	0.086
History of diabetes	1.67 (0.77-3.61)	0.191	1.40 (0.88-2.23)	0.155	1.63 (0.99-2.66)	0.054	1.86 (1.09-3.18)	0.023
History of dyslipidemia	1.05 (0.55-2.02)	0.873	1.24 (0.87-1.77)	0.230	1.23 (0.80-1.87)	0.342	1.57 (0.96-2.59)	0.074

Abbreviations: ICAS, Intracranial atherosclerotic stenosis; OXVASC, Oxford Vascular Study; CUHK-SR, the Chinese University of Hong Kong Stroke Registry; OR, odds ratio; CI, confidence interval.

eTable 6. Prevalence of ICAS in patients with ischemic stroke or TIA in Europe

Study	Country/ Region	Enrolment period	Sample size	Mean age, years	Male, %	IS/TIA	Cerebrovasc-ular imaging to define ICAS [a]	Cerebral vascular workups of the patients				Prevalence of ICAS, %		Modified NOS [b]
								TCD, % or n (%)	MRA, % or n (%)	CTA, % or n (%)	DSA, % or n (%)	Any ICAS	Symptomatic ICAS	
Weimar 2006 [1]	Germany	2000-2002	4,157	67	57	IS/TIA	TCD/MRA/ CTA/DSA	97	24	3	4	—	15	4
Holzer 2009 [2]	Germany	2000-2004	176	63	38	TIA	TCD	176 (100)	NA	NA	NA	12	—	2
Meseguer 2010 [3]	France	2003-2007	1,823	62	50	TIA	TCD/MRA/ CTA/HR-MRI	1,823 (100)	1,248 (69)	82 (5)	NA	9	4	3
Homburg 2011 [4]	Netherlands	—	786	62	56	IS/TIA	CTA	786 (100)	NA	NA	NA	10	3	3
Ovesen 2013 [5]	Denmark	2009-2011	652	67	56	IS/TIA	CTA	652 (100)	NA	NA	NA	7	0.5	3
von Sarnowski 2013 [6]	Pan-European countries	2007-2010	1,612	46	82	Young IS/TIA (age 18-55)	TCD	1,612 (100)	NA	NA	NA	20% in IS; 7% in TIA	14% in IS; 4% in TIA	4
Ssi-Yan-Kai 2013 [7]	France	2009-2011	129	64	57	Minor IS/ TIA	MRA	NA	129 (100)	NA	NA	—	12	2
Wolff 2014 [8]	France	2005-2010	159	37	51	Young IS (age 18-45)	TCD/MRA/ CTA/DSA	157 (99)	144 (91)	47 (30)	68 (44)	—	31	2
Logallo 2014 [9]	Norway	2010-2011	607	72	55	IS/TIA	TCCS/MRA/ CTA	TCCS: 607 (100)	533 (88)	227 (37)	NA	11	7	3

Tsivgoulis 2014 [10]	Greece	2009-2011	467	58	60	IS/TIA	TCD/MRA/ CTA/DSA	467 (100)	Only in those suspected of ICAS by TCD; numbers not reported			11	9	4
Baracchini 2016 [11]	Italy	2011-2013	1,134	71	59	IS	TCD/TCCS/ MRA/CTA	TCD/ TCCS: 1,134 (100)	844 (74)	290 (26)	113 (10)	9% (uncertain if it was any ICAS or symptomatic ICAS)		4
Sorgun 2017 [12]	Turkey	2012-2014	619	69	54	IS	MRA/CTA/ DSA	—	—	—	—	12	11	3
Hoshino 2018 [13]	France	2005-2008	403	62	74	IS	TCD/MRA/ CTA	399 (99)	289 (72)	53 (13)	NA	36	18	3

Abbreviations: ICAS, intracranial atherosclerotic stenosis or intracranial arterial stenosis of any etiology as defined in different studies; TIA, transient ischemic attack; IS, ischemic stroke; TCD, transcranial Doppler, MRA, magnetic resonance angiography; CTA, computed tomographic angiography; DSA, digital subtraction angiography; NOS, Newcastle-Ottawa Scale; HR-MRI, high-resolution magnetic resonance imaging; TCCS, transcranial color-coded Doppler sonography.

[a] ICAS was defined by velocity criteria in TCD or >/≥50% stenosis or occlusion in MRA/CTA/DSA, if not specified otherwise.

[b] With a total score of 0-5; scores of ≥3 or <3 respectively indicating low and high risk of bias.

"—" indicates unclear or not reported; "NA" indicates not applicable.

eTable 7. Prevalence of ICAS in patients with ischemic stroke or TIA in Asia

Study	Country/ Region	Enrolment period	Sample size	Mean age, years	Male, %	IS/TIA	Cerebrovascular imaging to define ICAS [a]	Cerebral vascular workups of the patients				Prevalence of ICAS, %		Modified NOS [c]
								TCD, n (%)	MRA, n (%)	CTA, n (%)	DSA, n (%)	Any ICAS	Symptomatic ICAS	
Lee 2002 [14]	Taiwan	1997-2001	170	—	71	Young IS/TIA (age 18-45)	DSA	NA	NA	NA	170 (100)	27	—	2
Shin 2005 [15]	South Korea	2000-2003	901	62	59	IS	MRA/DSA	NA	—	NA	—	28 [b]	22	3
Kim 2010 [16]	South Korea	2006-2009	1,012	64	63	IS/TIA	MRA	NA	1,012 (100)	NA	NA	27	—	3
Niu 2014 [17]	China	2007-2012	197	39	65	Young IS (age 15-49)	TCD/MRA/ CTA/DSA	160 (81)	MRA/CTA/DSA: 159 (81)			51	35	2
Shi 2014 [18]	China	2010-2012	351	47	72	Young IS (age≤55)	MRA/CTA/DSA	NA	315 (90)	24 (7)	12 (3)	35	—	3
Roy 2014 [19]	India	2007-2013	610	49	70	IS	Possibly MRA/CTA	NA	—	—	NA	—	36	3
Kiyohara 2014 [20]	Japan	2007-2012	693	69	62	TIA	MRA/CTA/DSA	NA	672 (97)	158 (23)	57 (8)	22		4
Ojha 2015 [21]	China	2007-2012	123	45	80	Young IS (age ≤50)	MRA	NA	98 (80)	NA	NA	38	—	2
Lee 2017 [22]	South Korea	2011-2016	516	68	52	IS	MRA	NA	516 (100)	NA	NA	43	—	3

Kim 2017 [23]	South Korea	2006-2009	1,081	64	62	IS/TIA	MRA/CTA/DSA	Mostly MRA (numbers not reported)				37	17	3
Park 2017 [24]	South Korea	2013-2013	9,506	66	61	Minor IS/ high-risk TIA	TCD/MRA/CTA/ DSA	6,990 (74)	7,494 (79)	3,255 (34)	945 (10)	—	25	4
Zhang 2019 [25]	China	2017-2018	207	62	66	IS	CTA	NA	NA	207 (100)	NA	65	—	3

Abbreviations: ICAS, intracranial atherosclerotic stenosis or intracranial arterial stenosis of any etiology as defined in different studies; TIA, transient ischemic attack; IS, ischemic stroke; TCD, transcranial Doppler, MRA, magnetic resonance angiography; CTA, computed tomographic angiography; DSA, digital subtraction angiography; NOS, Newcastle-Ottawa Scale.

[a] ICAS was defined by velocity criteria in TCD or >/≥50% stenosis or occlusion in MRA/CTA/DSA, if not specified otherwise.

[b] Prevalence of any ICAS was not reported in this article[15]. This number (28%) was retrieved from another article[26] reporting ICAS prevalence in this cohort but with a smaller sample size (n=512).

[c] With a total score of 0-5; scores of ≥3 or <3 respectively indicating low and high risk of bias.

"—" indicates unclear or not reported; "NA" indicates not applicable.

eTable 8. Prevalence of ICAS in patients with ischemic stroke or TIA in North and South America

Study	Country/ Region	Enrolment period	Sample size	Mean age, years	Male, %	IS/TIA	Cerebrovascul-ar imaging to define ICAS [a]	Cerebral vascular workups of the patients				Prevalence of ICAS, %		Modified NOS [c]
								TCD, % or n (%)	MRA, % or n (%)	CTA, % or n (%)	DSA, % or n (%)	Any ICAS	Symptomatic ICAS	
Sacco 1995 [27]	USA (multi-ethnic)	1990-1993	438	70	46	IS	TCD/MRA/ DSA [b]	75	—	NA	—	—	1% in Caucasians; 6% in African Americans; 11% in Hispanics	4
Wityk 1996 [28]	USA (multi-ethnic)	1993-1995	274	67	50	IS/TIA	TCD/MRA/ DSA	14	49	NA	14	24% in Caucasians; 22% in African Americans	9	3
Koch 2005 [29]	USA (Haitian)	1998-2002	126	60	52	IS	MRA/DSA	NA	MRA/DSA in 81 (64%)			—	9	3
Rincon 2009 [30]	USA (multi-ethnic)	1993-1997	714	60	37	First IS	TCD/MRA/ DSA [b]	54	18	NA	3	—	7	4
Lange 2018 [31]	Brazil	2012-2015	359	64	51	First IS	TCD/MRA/ CTA/DSA	315 (88)	MRA/CTA/DSA in 184 (51%)			—	5	3

Abbreviations: ICAS, intracranial atherosclerotic stenosis or intracranial arterial stenosis of any etiology as defined in different studies; TIA, transient ischemic attack; IS, ischemic stroke; TCD, transcranial Doppler, MRA, magnetic resonance angiography; CTA, computed tomographic angiography; DSA, digital subtraction angiography; NOS, Newcastle-Ottawa Scale.

[a] ICAS was defined by velocity criteria in TCD or >/≥50% stenosis or occlusion in MRA/CTA/DSA, if not specified otherwise.

[b] ICAS was defined by velocity criteria in TCD or >60% stenosis or occlusion in MRA/DSA in this study.

[c] With a total score of 0-5; scores of ≥3 or <3 respectively indicating low and high risk of bias.

"—" indicates unclear or not reported; "NA" indicates not applicable.

eTable 9. Prevalence of ICAS in patients with ischemic stroke or TIA in other countries/regions or worldwide

Study	Country/Region	Enrolment period	Sample size	Mean age, years	Male, %	IS/TIA	Cerebrovascular imaging to define ICAS [a]	Cerebral vascular workups of the patients				Prevalence of ICAS, %		Modified NOS [c]
								TCD, % or n (%)	MRA, % or n (%)	CTA, % or n (%)	DSA, n (%)	Any ICAS	Symptomatic ICAS	
TIAregistry.org project [32, 33]	World-wide	2009-2011	4,583	66	60	Minor IS/TIA	TCD/MRA/CTA	67	46	15	NA	14% overall; 20% in Japanese; 13% in non-Japanese [b]	—	4
Moustafa 2013 [34]	Egypt	2011	143	62	59	IS	MRA	NA	143 (100)	NA	NA	67	44	3

Abbreviations: ICAS, intracranial atherosclerotic stenosis or intracranial arterial stenosis of any etiology as defined in different studies; TIA, transient ischemic attack; IS, ischemic stroke; TCD, transcranial Doppler, MRA, magnetic resonance angiography; CTA, computed tomographic angiography; DSA, digital subtraction angiography; NOS, Newcastle-Ottawa Scale.

[a] ICAS was defined by velocity criteria in TCD or >/≥50% stenosis or occlusion in MRA/CTA/DSA, if not specified otherwise.

[b] 345 (7.5%) patients were Japanese; non-Japanese patients included European native (n = 3,317), Eastern Asian (n = 725), Middle or Proximal Eastern and Maghreb (n = 69), American Hispanic or Latino (n = 63), African American (n = 38), and other (n = 26).[33]

[c] With a total score of 0-5; scores of ≥3 or <3 respectively indicating low and high risk of bias.

"—" indicates unclear or not reported; "NA" indicates not applicable.

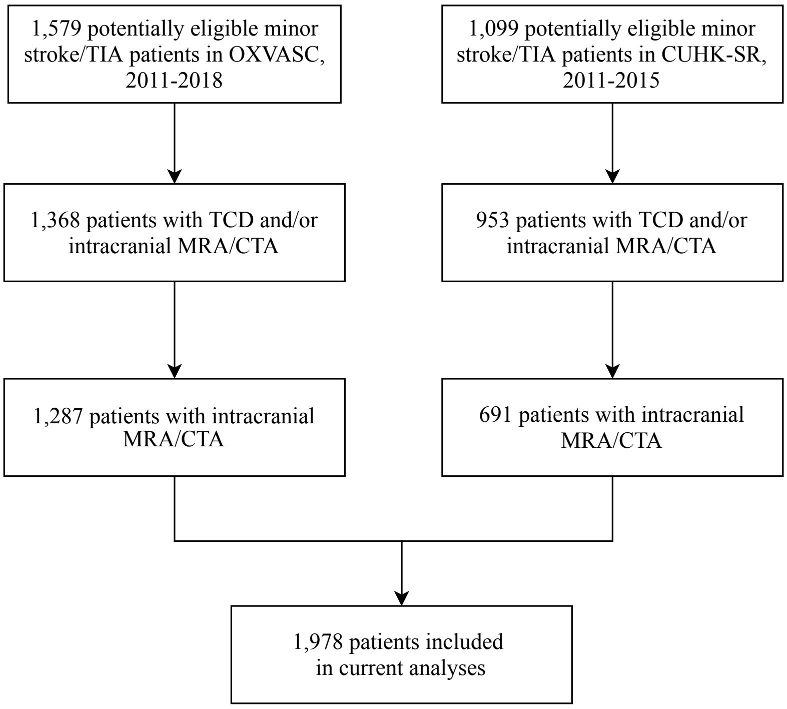

eFigure 1. Flow chart for patient screening in the current study.

Abbreviations: TIA, transient ischemic attack; OXVASC, Oxford Vascular Study; CUHK-SR, the Chinese University of Hong Kong Stroke Registry; TCD, transcranial Doppler; MRA, magnetic resonance angiography; CTA, computed tomography angiography.

Leng X, *et al. J Neurol Neurosurg Psychiatry* 2021; 92:590–597. doi: 10.1136/jnnp-2020-325630

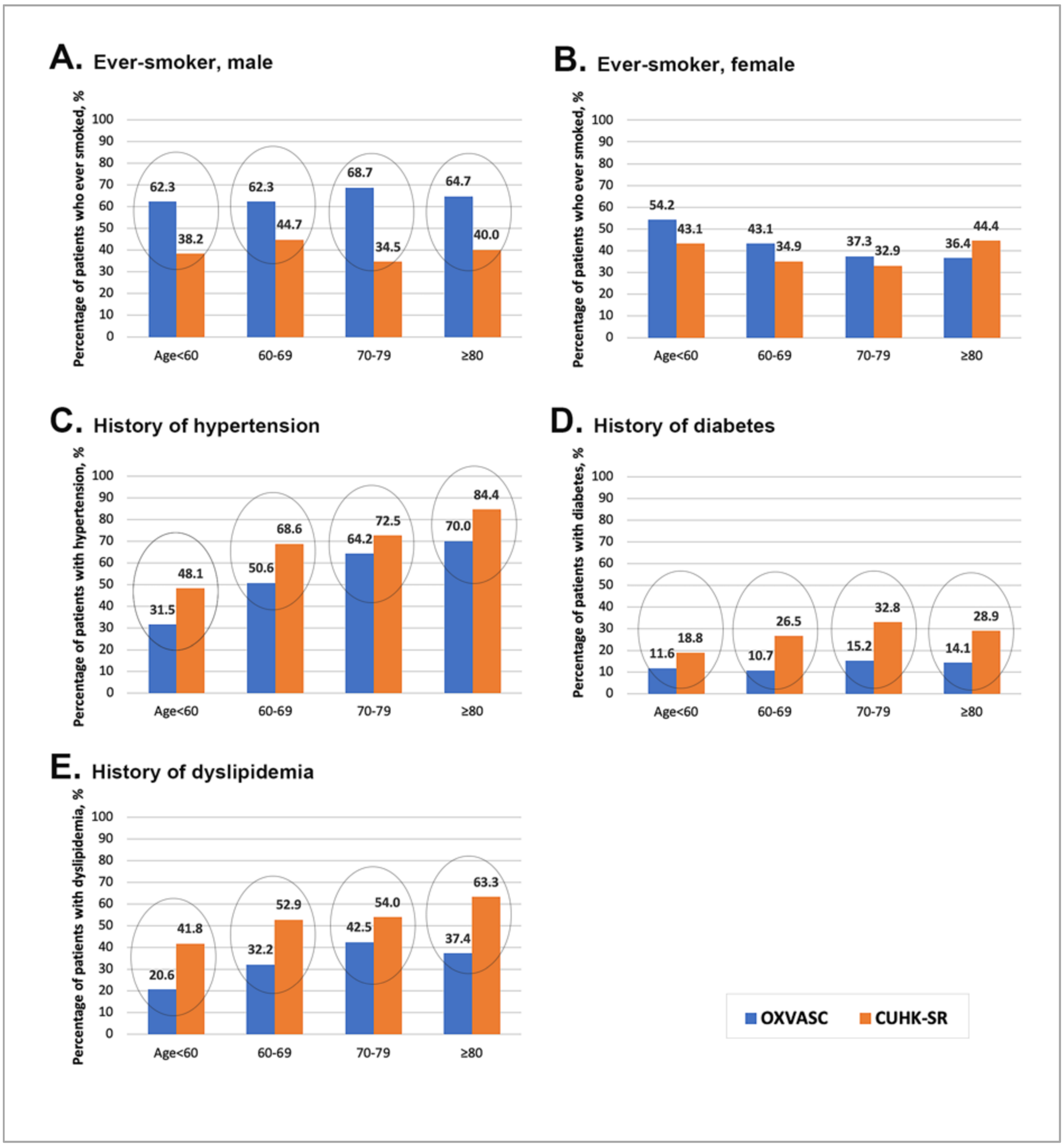

eFigure 2. Age-specified prevalence of vascular risk factors in the 2 cohorts.

A. More male patients in OXVASC had ever smoked than those in CUHK-SR in each age group.

B. The percentages of female patients who had ever smoked in the two cohorts were not significantly different in any age group.

C/D/E. More patients in CUHK-SR had histories of hypertension, diabetes and dyslipidemia than those in OXVASC, in each age group.

Circles indicate comparisons with p<0.05 for chi-square tests.

Abbreviations: OXVASC, Oxford Vascular Study; CUHK-SR, the Chinese University of Hong Kong Stroke Registry.

Leng X, *et al. J Neurol Neurosurg Psychiatry* 2021; 92:590–597. doi: 10.1136/jnnp-2020-325630

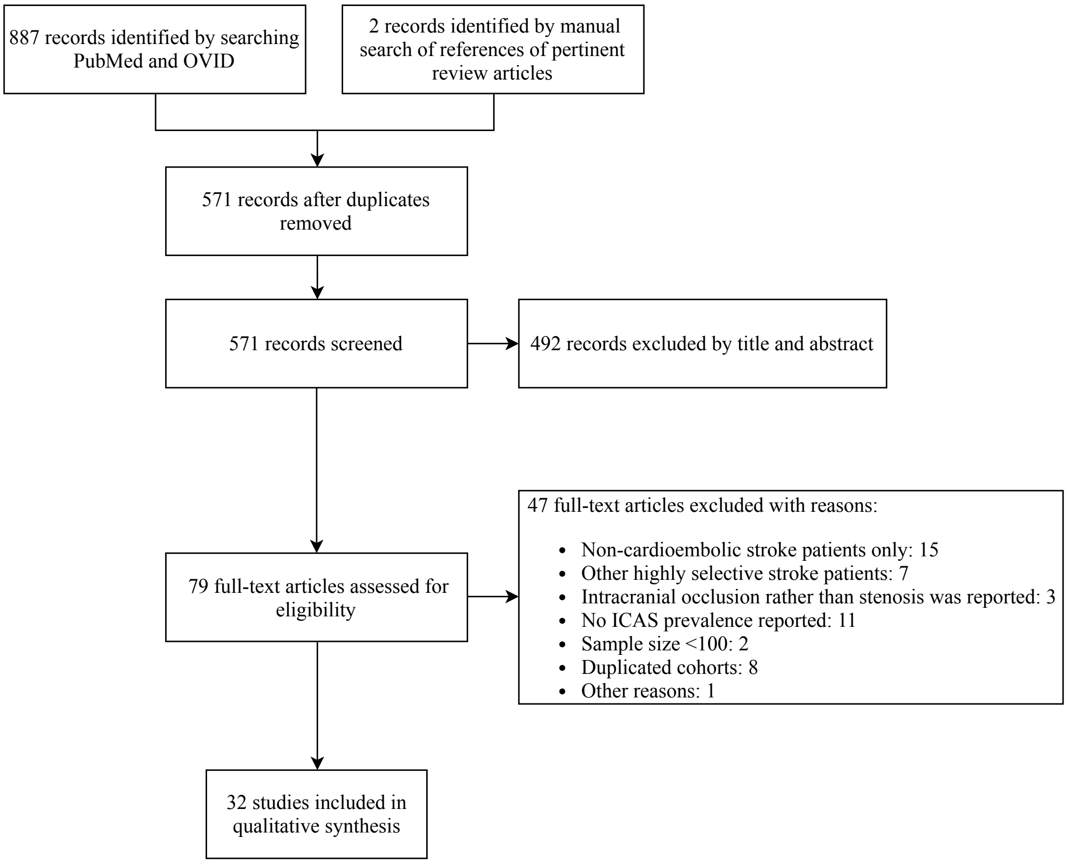

eFigure 3. Flow chart for study screening and selection in the systematic review.

Abbreviations: ICAS, intracranial atherosclerotic stenosis.

Appendix. Modified Newcastle-Ottawa Scale (NOS) [35, 36] for quality assessment of the primary studies.

(1) Representativeness of the sample:

1 point: Population contained a mixture of specialties or at multiple sites.

0 points: Population contained a single specialty at a single site.

(2) Sample size:

1 point: Sample size was greater than 200 participants.

0 points: Sample size was less than 200 participants or a convenience sample.

(3) Non-respondents:

1 point: Comparability between respondent and non-respondent characteristics was established, and the response rate was satisfactory.

0 points: The response rate was unsatisfactory, the comparability between respondents and non-respondents was unsatisfactory, or there was no description of the response rate or the characteristics of the responders and the non-responders.

(4) Ascertainment of intracranial arterial stenosis (ICAS):

1 point: Independent assessment using objective imaging methods.

0 points: No description/non-standard imaging methods used.

(5) Quality of descriptive statistics reporting:

1 point: Reported descriptive statistics to describe the population (*e.g.*, age, sex) with proper measures of dispersion (*e.g.*, standard deviation, standard error, range).

0 points: Descriptive statistics were not reported, were incomplete, or did not include proper measures of dispersion.

Legend: This scale, ranging from 0 to 5, assesses quality in several domains: sample representativeness and size, comparability between respondents and non-respondents, ascertainment of ICAS, and statistical quality. Studies were judged to be of low risk of bias (≥3 points) or high risk of bias (<3 points).

Leng X, *et al. J Neurol Neurosurg Psychiatry* 2021; 92:590–597. doi: 10.1136/jnnp-2020-325630

References in the supplemental materials

1. Weimar C, Goertler M, Harms L, et al. Distribution and outcome of symptomatic stenoses and occlusions in patients with acute cerebral ischemia. *Arch Neurol* 2006;63:1287-91.
2. Holzer K, Sadikovic S, Esposito L, et al. Transcranial Doppler ultrasonography predicts cardiovascular events after TIA. *BMC Med Imaging* 2009;9:13.
3. Meseguer E, Lavallee PC, Mazighi M, et al. Yield of systematic transcranial Doppler in patients with transient ischemic attack. *Ann Neurol* 2010;68:9-17.
4. Homburg PJ, Plas GJ, Rozie S, et al. Prevalence and calcification of intracranial arterial stenotic lesions as assessed with multidetector computed tomography angiography. *Stroke* 2011;42:1244-50.
5. Ovesen C, Abild A, Christensen AF, et al. Prevalence and long-term clinical significance of intracranial atherosclerosis after ischaemic stroke or transient ischaemic attack: a cohort study. *BMJ Open* 2013;3:e003724.
6. von Sarnowski B, Schminke U, Tatlisumak T, et al. Prevalence of stenoses and occlusions of brain-supplying arteries in young stroke patients. *Neurology* 2013;80:1287-94.
7. Ssi-Yan-Kai G, Nasr N, Faury A, et al. Intracranial artery stenosis or occlusion predicts ischemic recurrence after transient ischemic attack. *AJNR Am J Neuroradiol* 2013;34:185-90.
8. Wolff V, Armspach JP, Beaujeux R, et al. High frequency of intracranial arterial stenosis and cannabis Use in ischaemic stroke in the young. *Cerebrovasc Dis* 2014;37:438-43.
9. Logallo N, Naess H, Waje-Andreassen U, et al. Prevalence of intracranial stenosis in a Norwegian ischemic stroke population. *J Stroke Cerebrovasc Dis* 2014;23:1611-5.
10. Tsivgoulis G, Vadikolias K, Heliopoulos I, et al. Prevalence of symptomatic intracranial atherosclerosis in Caucasians: a prospective, multicenter, transcranial Doppler study. *J Neuroimaging* 2014;24:11-7.
11. Baracchini C, Anzola GP, Cenciarelli S, et al. Italian symptomatic intracranial atherosclerosis study (ISIDE) : A multicenter transcranial ultrasound evaluation. *Neurol Sci* 2016;37:1645-51.
12. Sorgun MH, Yilmaz V, Ulukan C, et al. Prevalence and prognosis of intracranial stenosis in acute ischemic stroke; a single center registry from Turkey. *Turk J Med Sci* 2017;47:1072-7.
13. Hoshino T, Sissani L, Labreuche J, et al. Prevalence of Systemic Atherosclerosis Burdens and Overlapping Stroke Etiologies and Their Associations With Long-term Vascular Prognosis in Stroke With Intracranial Atherosclerotic Disease. *JAMA Neurol* 2018;75:203-11.
14. Lee TH, Hsu WC, Chen CJ, et al. Etiologic study of young ischemic stroke in Taiwan. *Stroke* 2002;33:1950-5.
15. Shin DH, Lee PH, Bang OY. Mechanisms of recurrence in subtypes of ischemic stroke: a hospital-based follow-up study. *Arch Neurol* 2005;62:1232-7.
16. Kim BS, Jung HS, Bang OY, et al. Elevated serum lipoprotein(a) as a potential predictor for combined intracranial and extracranial artery stenosis in patients with ischemic stroke. *Atherosclerosis* 2010;212:682-8.
17. Niu JW, Gao S, Cui LY, et al. Intracranial atherosclerosis in Chinese young adult stroke patients. *J Stroke Cerebrovasc Dis* 2014;23:1519-23.
18. Shi Z, Zhang X, Chen Z, et al. Elevated thyroid autoantibodies and intracranial stenosis in stroke at an early age. *Int J Stroke* 2014;9:735-40.
19. Roy S, Das S, Danaboina R, et al. Association of E-selectin gene polymorphism (S128R) with ischemic stroke and stroke subtypes. *Inflammation* 2014;37:599-603.

20. Kiyohara T, Kamouchi M, Kumai Y, et al. ABCD3 and ABCD3-I scores are superior to ABCD2 score in the prediction of short- and long-term risks of stroke after transient ischemic attack. *Stroke* 2014;45:418-25.

21. Ojha R, Huang D, An H, et al. Distribution of ischemic infarction and stenosis of intra- and extracranial arteries in young Chinese patients with ischemic stroke. *BMC Cardiovasc Disord* 2015;15:158.

22. Lee SJ, Lee DG. Distribution of atherosclerotic stenosis determining early neurologic deterioration in acute ischemic stroke. *PLoS ONE* 2017;12

23. Kim BS, Chung PW, Park KY, et al. Burden of intracranial atherosclerosis is associated with long-term vascular outcome in patients with ischemic stroke. *Stroke* 2017;48:2819-26.

24. Park HK, Kim BJ, Han MK, et al. One-year outcomes after minor stroke or high-risk transient ischemic attack: Korean multicenter stroke registry analysis. *Stroke* 2017;48:2991-8.

25. Zhang S, Wang N, Chen L, et al. Serum Aldosterone Is Associated with Cerebral Artery Atherosclerosis and Calcification. *J Stroke Cerebrovasc Dis* 2019;28:523-30.

26. Bang OY, Kim JW, Lee JH, et al. Association of the metabolic syndrome with intracranial atherosclerotic stroke. *Neurology* 2005;65:296-8.

27. Sacco RL, Kargman DE, Gu Q, et al. Race-ethnicity and determinants of intracranial atherosclerotic cerebral infarction. The Northern Manhattan Stroke Study. *Stroke* 1995;26:14-20.

28. Wityk RJ, Lehman D, Klag M, et al. Race and sex differences in the distribution of cerebral atherosclerosis. *Stroke* 1996;27:1974-80.

29. Koch S, Pabon D, Rabinstein AA, et al. Stroke etiology among Haitians living in Miami. *Neuroepidemiology* 2005;25:192-5.

30. Rincon F, Sacco RL, Kranwinkel G, et al. Incidence and risk factors of intracranial atherosclerotic stroke: the Northern Manhattan Stroke Study. *Cerebrovasc Dis* 2009;28:65-71.

31. Lange MC, Ribas G, Scavasine V, et al. Stroke recurrence in the different subtypes of ischemic stroke. The importance of the intracranial disease. *Arq Neuropsiquiatr* 2018;76:649-53.

32. Amarenco P, Lavallée PC, Labreuche J, et al. One-year risk of stroke after transient ischemic attack or minor stroke. *N Engl J Med* 2016;374:1533-42.

33. Uchiyama S, Hoshino T, Sissani L, et al. Japanese versus non-Japanese patients with transient ischemic attack or minor stroke: subanalysis of TIA registry.org. *J Stroke Cerebrovasc Dis* 2019;28:2232-41.

34. Moustafa RR, Moneim AA, Salem HH, et al. Intracranial steno-occlusive arterial disease and its associations in Egyptian ischemic stroke patients. *Stroke* 2013;44:538-41.

35. Wells G, Shea B, O'Connell D, et al. The Newcastle-Ottawa Scale (NOS) for assessing the quality of nonrandomised studies in meta-analyses. Available at: http://www.ohri.ca/programs/clinical_epidemiology/oxford.asp. Accessed date: 14 December 2020.

36. Deng N, Zhang X, Zhao F, et al. Prevalence of lipohypertrophy in insulin-treated diabetes patients: A systematic review and meta-analysis. *J Diabetes Investig* 2017;9:536-43.
